# Beyond Bilateralism: Regional and Global Health Governance Implications of China–Pakistan Health Cooperation under the CPEC

**DOI:** 10.12669/pjms.42.8.20095

**Published:** 2026-08

**Authors:** Gao Jian, Xu Ziheng, Qu Qiumei

**Affiliations:** 1 Gao Jian Associate Professor, College of The Humanitiesm, Harbin Normal University, Harbin, China; 2 Xu Ziheng Associate Professor, School of Economics and Management, Harbin Normal University, Harbin, China; 3 Qu Qiumei Visiting Lecturer, Department of History & Pakistan Studies, University of the Punjab, Lahore, Pakistan

**Keywords:** Global Health Governance, South-South Cooperation, CPEC, Health Partnership

## Abstract

Global health governance faces challenges due to differences in philosophy, the complexity and political nature of the governance domain, and the uncertainty of funding sources. As an emerging economy, China will participate in global health governance through “South-South cooperation” and the “Belt and Road” Initiative. The China-Pakistan Economic Corridor (CPEC) serves as the hub and flagship project of the “Belt and Road” Initiative, while the China-Pakistan Health Corridor represents a specialized collaboration and extension of CPEC in the healthcare sector. Focusing on pharmaceuticals, disease prevention and control, and medical technology, it aims to improve the livelihoods of the Pakistani people and jointly advance the deepening of the All-Weather Strategic Partnership.

This study is part of the research project entitled “General Project of Philosophy and Social Sciences in Heilongjiang Province “A Study on the Pragmatic Motivation and Mechanism of ‘Sub-Prototypical Category Fuzzy Expressions’ in Modern Chinese” (22YYB250)”and “2022 Heilongjiang Province Postdoctoral Funding Project: Research on the Value of Marxist View of Literature and Art in the New Era(LBH-Z22205)”.

## INTRODUCTION

Some scholars have noted that global health governance faces challenges such as divergent governance philosophies and perceptions of interests, limitations of new stakeholders, an incomplete governance system, difficulties in allocating resources amid scarcity, and uncertainty regarding funding sources. As a member state of the global health governance framework, the attitudes, policies, and practices of the China-Pakistan Economic Corridor (CPEC) will, to a certain extent, influence or even determine the future direction of global health governance—this is the central significance of this study. This paper will analyze global health governance models and their shortcomings, explore how the China-Pakistan Economic Corridor (CPEC) can serve as a new model for global health governance, and discuss the challenges and limitations of CPEC as a global health governance model. Given the dynamic and evolving nature of global health governance and health diplomacy, future policy developments may affect the relevance and interpretation of the findings presented in this study.

## METHODOLOGY

This study focuses on regional and global health governance within the framework of health cooperation under the China-Pakistan Economic Corridor (CPEC). Combining qualitative literature analysis with comparative policy evaluation, the Official policy documents from Chinese government agencies like the State Council, the National Development and Reform Commission, the Ministry of Foreign Affairs, and the National Health Commission are among the trustworthy data and sources used in this study. The World Health Organization (WHO), the Planning Ministry of Pakistan, the Economic and Commercial Section of the Chinese Embassy in the Islamic Republic of Pakistan, and the National Agency for International Development Cooperation were among the international organizations whose reports and guidelines were incorporated. Institutions like Jianpei Technology and the China Association for Science and Technology contributed further scholarly study and policy publications. Relevant information was obtained from media sources like Today Pakistan, China Daily, Xinhua News Agency, and China Economic Net.

### Global Health Governance Models:

Global health governance has gradually evolved into a multi-tiered international cooperation mechanism centered on the World Health Organization (WHO) and supported by other health-related organizations. Through established norms and mechanisms, this framework promotes coordination and cooperation among nations, international organizations, and other non-state actors to address global health challenges and safeguard the health and well-being of all humanity.^1^

Although a global health governance mechanism centered on the WHO has largely taken shape, the WHO has long been unable to independently allocate medical resources due to chronic underfunding; furthermore, constrained by political issues, effective cooperation is often difficult to achieve; Furthermore, the WHO’s reliance on a limited medical-technical approach prevents it from addressing the complexity of public health issues. For these reasons, the WHO is generally unable to truly assume a leadership role.

This system suffers from a severe lack of capacity-building assistance when addressing new pandemics such as COVID-19. Specifically, the WHO’s disease-specific assistance programs have long lead times and their effectiveness is difficult to measure; moreover, the effectiveness of the WHO’s pandemic response mechanisms remains inadequate. Funding for the WHO’s response to the COVID-19 pandemic remains extremely limited, and there is a severe shortage of necessary resources.^2^

This is also reflected in the WHO’s lack of sufficient political support. Specifically, in the face of a sudden public health emergency such as the pandemic, the WHO lacks legal authority, and few countries have heeded its recommendations for temporary travel and trade restrictions. The WHO also lacks a unified public health reporting mechanism.

When addressing chronic noncommunicable diseases (such as cardiovascular disease, cancer, chronic respiratory diseases, and diabetes) and tackling issues of unequal resource distribution—such as the significant disparities in the allocation of medical facilities, healthcare personnel, and equipment between urban and rural areas and across regions, which lead to difficulties in accessing care and high healthcare costs, with high-quality resources concentrated primarily in large cities—efforts often face limitations such as slow response times, funding shortages, and a lack of enforceability. Developed countries have snapped up the majority of high-quality vaccines through advance purchase agreements, leaving low and middle-income countries unable to obtain the vaccines they need.^3^

Some developed countries in the West view bioterrorism as a priority for global health governance, rather than the diseases that have caused massive global mortality over the past few decades.^4^ This has led to a loss of trust in the World Health Organization among relatively less developed countries, resulting in a significant decline in participation in global health governance.

The limitations on the influence of certain member states in global health governance as they transition from aid recipients to active participants. Some Western countries encourage and promote private-sector participation in global development aid. Private philanthropic foundations are the most significant force and form of private-sector engagement in global health governance.^5^

While addressing diseases and establishing a global public health system are shared goals of global health governance, in practice, certain Western countries have primarily focused their limited resources on the prevention and treatment of specific diseases, primarily AIDS, malaria, and tuberculosis. In reality, due to the lack of robust healthcare systems, a large number of mothers, newborns, and children in developing countries die each year from health issues such as chronic diseases—including ischemic heart disease, diabetes, and cancer.

For global health governance to be effective, it must address the divergent interests of developed and underdeveloped nations. It must allocate limited funds to health initiatives in developed nations while simultaneously ensuring the prevention and control of both infectious and non-communicable diseases in underdeveloped regions. Only then can this mechanism fulfill its intended purpose.

### CPEC as a New Model for Global Health Governance:

Achieving health for all is a shared aspiration of humanity. Against the backdrop of increasingly transnational and complex global public health risks, China and Pakistan have actively worked to establish the China-Pakistan Health Corridor in recent years. From dispatching medical teams and training personnel, to preventing and controlling infectious diseases and providing health assistance, to promoting traditional Chinese medicine and signing cooperation agreements… a cooperative framework centered on development-oriented approaches and capacity building has gradually taken shape. This model is evolving from bilateral cooperation between China and Pakistan into a public health cooperation mechanism at the regional and even global levels. China has signed health cooperation agreements with more than 160 countries and international organizations, driving health cooperation from project-based assistance toward institutionalized governance. Through concrete actions, China is improving the global public health governance system and making significant contributions to advancing the building of a global community of health for all.

In practical terms, from infectious disease prevention and control, and health assistance, to talent development and the promotion of traditional Chinese medicine, health exchanges and cooperation between China and Pakistan have continued to deepen, gradually forming replicable and scalable development experiences. The fruits of this cooperation are spreading to the Belt and Road regions and beyond.

In January 2017, China and the World Health Organization (WHO) signed a Memorandum of Understanding on cooperation in the health sector under the Belt and Road Initiative. This marked the formal integration of health cooperation between China and Pakistan—and indeed across the Belt and Road Initiative—into the global health governance framework. This landmark document signifies that China’s practical cooperation with the WHO has expanded to the Belt and Road participating countries, the regional level, and the global stage.

In short, the China-Pakistan Health Corridor is not only a bilateral cooperation mechanism but has also gradually evolved into an important platform for promoting global health equity and capacity balance; its defining characteristic is that of a “development-oriented new model of global health governance.”

### The Three Key Features of the CPEC Model:

The China-Pakistan Health Corridor has gradually evolved into a unique model that differs from traditional approaches to global health governance. Its core characteristics are primarily reflected in the following aspects.

### Integration of Medical Systems:

By integrating the primary healthcare systems of China and Pakistan, the China-led telemedicine program in Pakistan has achieved significant breakthroughs, particularly in bridging the gap between primary and secondary healthcare systems. Through online medical services, residents—especially those in remote areas—are now able to access timely disease prevention services.^6^

### Technology-enabled System Embedding:

Against the backdrop of the Belt and Road Initiative’s deepening development, the construction of the Pakistan-China Economic Corridor has entered a new phase of high-quality development. Strengthening exchanges and cooperation between Pakistan and China in the field of healthcare is of great significance for advancing the building of a Pakistan-China community with a shared future and a health community. In particular, the Pakistan-China Health Corridor comprises medical institutions, nursing facilities, and auxiliary healthcare providers; through the use of artificial intelligence, virtual reality, and big data, it enables Pakistan to benefit from China’s achievements in medical development.

### Capacity Building and Localization:

it is essential to strengthen local talent development by leveraging Pakistan’s needs and the resources and expertise of relevant medical organizations and academic institutions. This should involve cultivating professionals in preventive medicine, health inspection, and quarantine through diverse methods and channels to enhance the resilience of the China-Pakistan infectious disease prevention and control system.^7^

The traditional model and the CPEC model has been comprehensively illustrated in Table-I.

**Table-I:** A Comparison of the Traditional Model and the CPEC Model.

Dimension	Traditional Model	CPEC Model
Actors	led primarily by Western countries and international organizations (such as the WHO and the World Bank)	South-South cooperation centered on China and Pakistanis largely driven by bilateral or regional initiatives
Governance Structure	Led by multilateral mechanisms, but hierarchical in nature	Bilateral + Embedded in National Development Strategies: A More Flexible Structure(hybrid & embedded)
Approach	primarily aid-based	co-development & co-construction
Financing	Reliance on donor countries and international funds	Government Investment + Project Financing + Long-Term Partnership Mechanism
Objectives	Focusing on disease-specific control	With a focus on strengthening the health system
lntervention Type	Vertical programs (such as those for HIV/AIDS and malaria)	Horizontal integration (e.g., infrastructure + healthcare)
Time Horizon	Short-term project-oriented	long-term development-oriented
Capacity Building	Relatively limited, reliant on external support	Emphasizing technology transfer and local capacity building
Technology Transfer	Fewer, with technical barriers	Systematic Technology Transfer and Training Mechanism
Localization	Lower, reliant on external experts	Emphasizing localization and self-reliance
Institutionalization	Project-based approach, with weak institutional integration	Deeply embedded in national policies and bilateral mechanisms
Sustainability	Lower (discontinued upon project completion)	Higher (supported by institutional frameworks and capacity building)
Equity	There is a problem of inequality between the north and the south	Emphasizing cooperation among developing countries (South-South cooperation)
Crisis Response	Relies on international coordination, resulting in a slower response	Bilateral coordination is more efficient (e.g., pandemic aid)
Spillover Effects	Weak, limited to the scope of the project	Relatively strong, with the potential to spread regionally
Governance Philosophy	The donor-recipient paradigm	The Logic of Development Cooperation (Partnership and Mutual Benefit)
Norms & Discourse	Western-dominated rules and standards	Emerging governance norms (South-led governance norms)
Replicability	Highly standardized, but with limited adaptability	Highly context-dependent (context-dependent) Requires adjustment and copying

***Source:*** Author’s own elaboration..

## CONCLUSIONS

Health cooperation under the framework of the China-Pakistan Economic Corridor (CPEC) is shifting from bilateral cooperation between the two countries to broader global participation. The scope of cooperation is expanding from infrastructure construction and the transfer of medical technology to the training of medical personnel and the exchange of talent. The development philosophy is evolving from a development-oriented approach toward achieving synergistic win-win outcomes. Health cooperation under the CPEC provides replicable experiences for global health governance. In the future, the trajectory of health cooperation under the CPEC will become increasingly clear and will garner greater attention from more countries around the world.
